# Peripheral Neuroergonomics – An Elegant Way to Improve Human-Robot Interaction?

**DOI:** 10.3389/fnbot.2021.691508

**Published:** 2021-08-20

**Authors:** Alessandro Del Vecchio, Claudio Castellini, Philipp Beckerle

**Affiliations:** ^1^Department of Artificial Intelligence in Biomedical Engineering, Faculty of Engineering, Friedrich-Alexander Universität Erlangen-Nürnberg, Erlangen, Germany; ^2^Institute of Robotics and Mechatronics, DLR German Aerospace Center, Weßling, Germany; ^3^Chair of Autonomous Systems and Mechatronics, Department of Electrical Engineering and Department of Artificial Intelligence in Biomedical Engineering, Faculty of Engineering, Friedrich-Alexander Universität Erlangen-Nürnberg, Erlangen, Germany; ^4^Institute for Mechatronic Systems, Mechanical Engineering, Technical University of Darmstadt, Darmstadt, Germany

**Keywords:** peripheral interfaces, myography, user experience, neuroergonomics, human-robot interaction

## 1. Introduction

The day seems not too far away, in which robots will be an active part of our daily life, just like electric appliances already are. Hence, there is an increasing need for paradigms, tools, and techniques to design proper human-robot interaction in a human-centered fashion (Beckerle et al., 2017). To this end, appropriate Human-Machine Interfaces (HMIs) are required, and there is a growing body of research showing how the Peripheral Nervous System (PNS) might be the ideal channel through which this interaction could proficiently happen.

During daily motor tasks such as grasping, walking, or speaking, the central nervous system (CNS) recruits a number of α-motoneurons in the ventral horn of the spinal cord and modulates the rate at which they discharge action potentials. The α-motoneurons are further modulated by supraspinal, afferent volleys and intrinsic motoneuron properties (Heckman et al., 2005; Enoka, 2008). The motoneuronal axonal action potentials are transformed into forces by a group of muscle fibers (the muscle unit) innervated by one axon. The muscle unit and the motoneuron form the final ensemble of all motor actions, the so-called *motor unit*. Translating neural commands into muscular forces, (spinal) motor units represent a promising interface to the CNS. However, there are some physiological constraints of motor control that must be taken into account by robotic applications.

**Figure 1:**
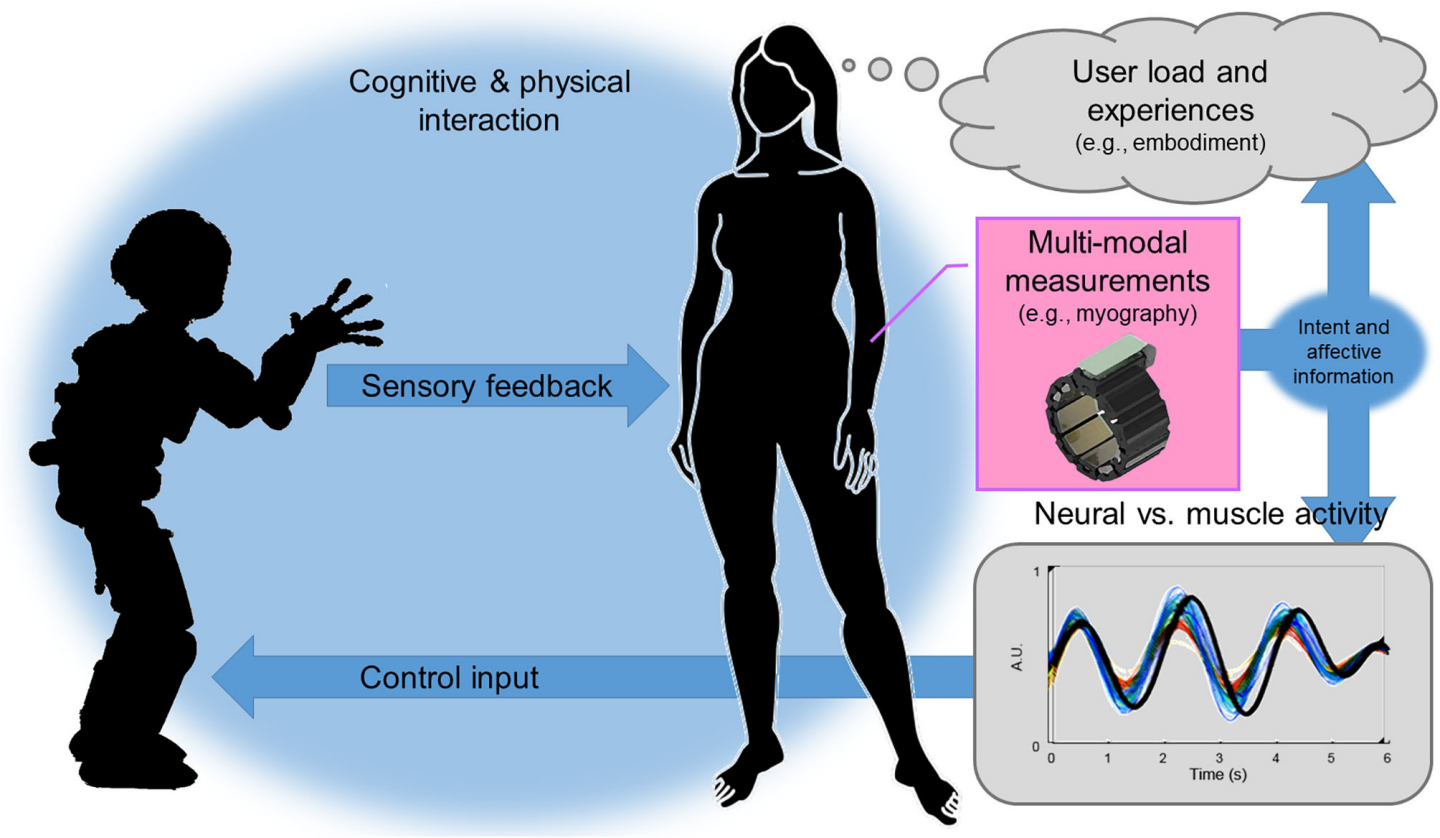
Peripheral-Nervous-System–Machine Interfaces (PNS-MIs) in human-robot interaction: PNS data facilitates simultaneous intent recognition and online experience evaluation. This fosters novel sensorimotor interaction paradigms, sheds light on human behavior and reactions, and thereby opens up new directions for human-robot interaction.

In this opinion paper, we claim that better user experience would lead to more intuitive control and tighter human-robot interaction or even human-machine integration and vice-versa (see Figure 1).

Using PNS data for intent detection as well as for online assessment of user experience renders such interfaces technically promising and a tool to understand human behaviors and reactions (Beckerle et al., 2019). To improve on this, we discuss developments in intent detection and user feedback and user feedback emphasizing on anthropomorphic systems, which are directly controlled by humans, e.g., prostheses and teleoperation, and aiming to create novel sensorimotor paradigms.

## 2. Peripheral-Nervous-System–Machine Interfaces (PNS-MIs)

Interfaces for controlling anthropomorphic robotic systems, e.g., HMIs for self-powered prostheses, cannot function like a joystick or a touch-screen for instance, since the user cannot physically operate such devices. These HMIs must rather resort to interpreting the user's intent based on signals the user is able to produce - usually, relevant biological signals related to the intended muscle activation (Beckerle et al., 2019). Surface electromyography (Merletti et al., 2011) is a primary example, although different kinds of signals are currently being explored, e.g., tactile information (Beckerle et al., 2018, 2019) and also promising for other applications such as anthropomorphic teleoperation (Nostadt et al., 2020) or teaching collaborative robots (Cansev et al., 2021).

In principle, all signals generated by the users can and should be used to interpret their intent, but clearly, the optimal choice of signals and sensors depends on a tradeoff involving several factors. This includes, e.g., how well the sensors can be worn (ergonomy), how expensive the processing is (both economically and computationally), and how invasive their setup is. Furthermore, intent detection does not necessarily coincide with classification of signal patterns; rather, it's the ability to provide the user a seamless control experience based upon such signals, e.g., using regression instead of classification.

### 2.1. The Pros and Cons of CNS/PNS-MIs

Broadly speaking, signals related to movement and muscle activation can be classified according to whether they are recorded from the CNS or the PNS (Castellini et al., 2014). HMIs relying on CNS signals include brain-machine interfaces using surface electroencephalography as well as electrocorticography with direct implants on the motor cortex and spinal implants (Micera et al., 2010), or by decoding spinal motoneurons from high-density EMG signals (Farina et al., 2017; Del Vecchio and Farina, 2019). PNS-based HMIs, on the contrary, are those using signals recorded from the limbs, either invasively or non-invasively, e.g., implanted electromyography and direct connections to peripheral nerves vs. surface electromyography, force- and magneto-myography (Fang et al., 2015). Although EMG interfaces are placed in the periphery (muscles) the signal carried by the electrical activity generated by muscle fibers is in a one-to-one relation with spinal motoneurons. Moreover, minimally invasive approaches like local tomography of the limbs, which entail no surgery but indeed the injection of energy into the body, exist (Sierra González and Castellini, 2013; Gibas et al., 2019).

Given the extreme density of neural cells found in the CNS, most signals useful for robotic control to be potentially found in it are physically unavailable for direct inspection, unless one resorts to very invasive methods, e.g., the Braingate (Hochberg et al., 2012). Non-invasive methods, on the other hand, strongly suffer from cross-talk: the main problem is to tell signals pertaining to the intent under examination from “all the rest.” Surface electroencephalography, for instance, poses extremely complex problems to interpret and discern the neural firing patterns of interest, since each sensor can only record potentials from a large pool of neurons. Accordingly, damping and distorting effects due to skull bone tissue complicate pattern recognition (Lazarou et al., 2018). As opposed to that, an excellent signal-to-noise ratio can be obtained at the price of getting in contact with the cerebral cortex or the spinal cells (Hochberg et al., 2012).

PNS-based systems, on the other hand, can use better separated and physiologically relevant signals, naturally enforced by the anatomical branching of nerves and neurons as they depart from the brain, brainstem, and spinal cord. If one is interested in detecting the intent to move, exert forces and torques, and/or activate one's own muscles, then detecting such activity from the PNS appears to be a better choice especially if non-invasiveness is desired (Castellini et al., 2014). On top of this, if minimal invasiveness is permitted or desired, PNS approaches are probably even the best choice nowadays. Ultrasound scanning and electromyographic sensors implanted during osseointegration (Ortiz-Catalan et al., 2014) or injected into the muscles (Becerra-Fajardo and Ivorra, 2019) offer high signal-to-noise ratios while entailing rather low risk.

### 2.2. Improving on PNS-MIs

It has been known to physiologists for the last three decades that the neural activation that is transmitted by the motoneuron is delayed by the muscle tissue over a large range of values, from roughly 50 to more than 200 ms (Partridge, 1965; Baldissera et al., 1998). During fast motor tasks the nervous system compensates this delay by increasing the motoneuron firing frequency and the delay between the recruitment of successive motor units. Therefore, the CNS tunes this delay dynamically. Previous experiments in animal preparations demonstrated that changes in stimulation frequency alters the delay between the myoelectrical signal and the force produced by the muscle tissues in a very large range (Partridge, 1965; Baldissera et al., 1998). Recently, by decoding the activity of a large population of motoneurons during contraction at different speeds, we also found that the human nervous system modulates such delays in a very broad range (50–250 ms for hand and leg muscles Del Vecchio et al., 2018).

In virtually all prosthetic applications, however, this delay is fixed (Farina et al., 2014) yielding devices that do not follow the physiological modulation during natural processes like muscle fatigue (Zhou et al., 1998), adaptation of contraction speed (Del Vecchio et al., 2018), and muscular force output (Del Vecchio et al., 2018). Still, neuroergonomics should indeed translate these basic physiological findings into novel interface designs and, potentially, prosthetic applications for improving human robot-interactions. One potential solution to overcome this limitation is to decode surface EMG in real-time. We have previously shown that it is possible to retrieve the motoneuron discharge timings with delays smaller than 2 ms (Glaser et al., 2013; Barsakcioglu and Farina, 2018; Chen et al., 2020; Ting et al., 2021). Moreover, the potential to identify individual motor unit discharge times allows to label each motor unit to its unique motor space, e.g., encoding flexion/extension or which digit. Therefore, classification of EMG activity can be performed in a highly accurate way by associating each motor unit to its specific spatiotemporal space, as shown in a spinal cord injury case (Ting et al., 2021).

### 2.3. Considering User Experience Through PNS-MIs

Recent research outlines that PNS-MIs also have potential in directly assessing user experience going beyond established psychometric and physiological methods. An interesting example is the embodiment of robotic systems such as prostheses or teleoperation systems (Beckerle et al., 2019; Nostadt et al., 2020): the embodiment of artificial limbs can be assessed through surveying subjective experience with questionnaires (Longo et al., 2008), objective behavioral measures (e.g., proprioceptive drift), or (neuro)physiology (Christ and Reiner, 2014). This effect was also shown for artificial limbs with myoelectric control (Romano et al., 2015; Sato et al., 2018), but we might ask ourselves whether myoelectric measurements could also be used to analyze neuroergonomics of interaction with such devices. Recent work by Preatoni et al. for instance (Preatoni et al., 2021) indicates that proper sensory feedback makes a leg prosthesis feel lighter.

For patients suffering from stroke, the experience of device embodiment seems to have similar influence on electromyographic activity as for other physiological measures, i.e., electrodermal activity and skin temperature (Llorens et al., 2017). While, Tsuji et al. (2013) even report subjective survey results to be better represented by electromyography than by electrodermal activity, (Llorens et al., 2017) state that interactions between their subjective and neurophysiological results were inconclusive. Besides embodiment, the perception of pleasantness of affective touch can be related to electromyographic as well as to electrodermal measurements (Ree et al., 2019). This is very interesting since providing affective information through touch was shown to increase the embodiment of artificial limbs (Crucianelli et al., 2013, 2018; van Stralen et al., 2014) and, hence, appears worth considering in human-robot interaction (Beckerle et al., 2018).

Although myographic activity was measured at different sites, i.e., hand and face (Tsuji et al., 2013; Llorens et al., 2017; Ree et al., 2019), considering it in the assessment of user experience seems promising. We have ourselves recently put forward the potential connection between control based upon muscle activation and action schemes in the sense developed by Piaget (Piaget, 1950). Here, a proper PNS-MI could foster the creation of novel circular reactions, leading to embodiment as a natural consequence (Bettoni and Castellini, 2021). The factors influencing the effect remain unexplored so far. Understanding and shaping these interactions might be supported by multimodal data from an interface integrating myography with other physiological data, e.g., electrodermal activity or heart rate.

## 3. Discussion

With this position paper, we advocate peripheral neuroergonomics as an *elegant* way to improve HRI. Non-invasively interfacing the peripheral nervous system seems to provide very good interpretability and is currently advantageous over CNS-based interfaces, which outline higher invasiveness as well. Moreover, peripheral interfaces can augment or complement other modalities such as eye-tracking and electroencephalography to improve the recognition of user intentd and cognitive status. Generally, we expect considering neuromechanical insights in novel interfaces designs to foster improved HRI characteristics of robotic systems and devices. An accurate closed-loop control of the neuromechanical delays matching the physiological pathways would likely improve sensorimotor interactions. In addition, peripheral neural information can complement psychometric and physiological methods to assess user experience, which indicates that integrating myographic assessment in multimodal PNS-MIs would bring the neuroergonomics of human-robot interaction to a new level of quality.

## Author Contributions

PB coordinated its development as well as the integration of individual contributions. All authors conceptualized the structure, contributed content, perspectives, and references as well as discussed and revised the manuscript.

## Conflict of Interest

The authors declare that the research was conducted in the absence of any commercial or financial relationships that could be construed as a potential conflict of interest.

## Publisher's Note

All claims expressed in this article are solely those of the authors and do not necessarily represent those of their affiliated organizations, or those of the publisher, the editors and the reviewers. Any product that may be evaluated in this article, or claim that may be made by its manufacturer, is not guaranteed or endorsed by the publisher.
